# Identifying contributory risk factors for neck pain in fast jet aircrew: a prospective cohort study

**DOI:** 10.1007/s00420-025-02162-7

**Published:** 2025-08-14

**Authors:** James B. Wallace, Peter G. Osmotherly, Tim J. Gabbett, Wayne Spratford, Phil M. Newman

**Affiliations:** 1 University of Canberra Research Institute for Sport and Exercise (UCRISE), Bruce, ACT Australia; 2 Ethos Health, Newcastle, NSW Australia; 3 School of Health Sciences, The University of Newcastle, Callaghan, NSW Australia; 4 Gabbett Performance Solutions, Brisbane, QLD Australia

**Keywords:** Injury, Military personnel, Preventative medicine, Musculoskeletal system, Occupational health services

## Abstract

**Purpose:**

Fast jet aircrew (FJA – aka fighter pilots, fighter aircrew) commonly suffer musculoskeletal complaints, particularly of the neck, which degrades operational capability and workforce health. Establishing injury aetiology is a prerequisite for developing effective preventative interventions. Our objective was to examine the aetiology of neck-related musculoskeletal complaint episodes (NRMCEs) among FJA across a range measures including physical capacity, psycho-social recovery-stress states, workload measures, and strength and conditioning (S&C) participation.

**Methods:**

279 Royal Australian Air Force (RAAF) FJA were followed over four 5-month reporting periods. Forty-four baseline measures and 26 weekly measures were analysed for their effect on weekly risk of NRMCEs. Mixed-effects logistic regression was used, potential confounders were adjusted for, and non-linear relationships were examined.

**Results:**

320 new NRMCEs were included with mean weekly prevalence of 4.1% (SD 2.3) and mean 5-month reporting period prevalence of 22.4% (range 15.3–28.5%). Previous neck pain, higher worry scores, larger acute flying workloads, more weekly flights, and larger acute and chronic S&C workloads, were factors identified to increase weekly risk of NRMCE. Significant non-linear effects were identified for chronic flying workloads, acute and chronic sleep quality scores, and absolute isometric strength of neck rotation and trunk flexion. Demographics, flying history, anthropometry measures, neck and trunk range of motion, and S&C participation, however, did not affect weekly risk of new NRMCE.

**Conclusion:**

These findings provide crucial support for the design of targeted prevention programs for FJA, ensuring they are both effective and efficient, which will in turn optimise operational capability.

**Supplementary Information:**

The online version contains supplementary material available at 10.1007/s00420-025-02162-7.

## Introduction

Fast jet operation is integral to attaining and maintaining air superiority, which is critical to battlefield outcome (Saunders and Souva 2020). Fast jets (e.g. F/A-18, Eurofighter, and F-35) are highly capable aircraft, being very agile, powerful, and equipped with advanced weapons and sensor systems (Newman 2016). Fast jet aircrew (FJA - also known as fighter pilots/fighter aircrew) are thus exposed to arduous cognitive and physical demands, including: repetitive anti-G straining manoeuvres, repetitive neck movements into awkward positions, and, supporting their head, helmet, and helmet-mounted equipment under high gravitational forces (Balldin 1986; Green and Brown 2004).

Unsurprisingly, FJA suffer high rates of musculoskeletal complaints (an umbrella term encompassing terms such as pain and injury without delineations around exposure, onset, or diagnosis), particularly effecting the neck (Wallace et al. 2023). These often impair operational capability by: reducing flying performance (Wallace et al. 2023) and availability (Netto et al. 2011; Wagstaff et al. 2012), dropout from costly training, and early career termination (Sovelius et al. 2022). Effective and efficient preventative measures are needed to prevent such impacts upon operational capability and FJA health.

Establishing the aetiology of musculoskeletal complaints is a prerequisite for developing and implementing effective and efficient preventative interventions (van Mechelen et al. 1992). However, research investigating aetiology of neck-related musculoskeletal complaint among FJA has been hampered by the complexities of researching FJA including: security, limited access, small populations, busy schedules, frequent travel, and reluctance to provide medical information (Wallace et al. 2021). Previous research among FJA has identified there is a need for studies that are prospective, use agreed definitions and validated surveillance methods, minimise recall-bias, undertake multivariable analyses, and have larger sample sizes (Wallace et al. 2021). Furthermore, investigations of the aetiology of musculoskeletal complaints in other populations have identified a range of methods and factors that may have good application with FJA, such as workload monitoring (Drew and Finch 2016; Windt and Gabbett 2017) and psycho-social recovery-stress states (‘wellbeing’) (Jones et al. 2017; Saw et al. 2016).

The purpose of this study was to examine potential contributory risk factors for neck-related musculoskeletal complaint episodes (NRMCEs) among FJA across demographic, physical capacity, psycho-social recovery-stress states, and workload measures using a mix of physical testing, review of existing data capture systems, and self-report measures. Specific questions included: does flying workload measured using session rate of perceived exertion (sRPE) affect NRMCE risk; are there specific strength-related variables that affect NRMCE risk; are there demographic and role-specific features that affect NRMCE risk; do psycho-social recovery-stress states affect NRMCE risk; and, does FJA-targeted strength and conditioning (S&C) participation effect NRMCE risk? The findings will assist the design and refinement of targeted preventative interventions among FJA.

## Methods

### Study design

Royal Australian Air Force (RAAF) FJA were followed during four consecutive 5-month reporting periods during 2019–2020. Reporting periods aligned to flying/training schedules, and accounted for most FJA posting cycles. Informed consent was sought from FJA, and those posting into units afterwards were also invited to participate. Approval was provided by Joint Health Command Low Risk Ethics Panel (reference 17–018) with University of Canberra Human Research Ethics Panel (reference 20170089) providing cross-institutional approval. This study is reported using the STROBE (Von Elm et al. 2007).

### Participants

A convenience sample was used where all RAAF FJA were invited to participate if they were posted to a fast jet unit (i.e. Hawk-127, F/A-18, F/A-18 F, EA/18-G, F-35) within the study period. To account for FJA that posted in/out of units partway through a reporting period, only weeks that they were present/posted in a relevant unit was considered in analyses. To align with our previous work (Wallace et al. 2023), FJA present for less than half a reporting period were excluded.

### Data collection and definitions

#### Outcome definition

NRMCEs were captured using the validated University of Canberra Fast Jet Aircrew Musculoskeletal Questionnaire (UC-FJAMQ) (Wallace et al. 2022, 2023). FJA were asked whether they had experienced a musculoskeletal complaint (‘*refers to pain*,* ache*,* discomfort*,* stiffness*,* or tingling or numbness which may be experienced anywhere in your body’*) in the previous 7-days, and if so, they would detail its location, severity, duration, onset, and impact. A new NRMCE was the outcome of interest. FJA completed the UC-FJAMQ weekly using Smartabase smartphone app and online software (Fusion Sport, Australia), with weekly reminders (email and smartphone push-notifications) sent.

#### Baseline data collection

At the start of each reporting period, FJA were asked to undertake baseline testing. A questionnaire captured: age, flying role, cockpit role, total fast jet flying hours, and previous neck pain. FJA rated their personal ‘worry’ on 0–10 scale (0 = not worried at all, and 10 = extremely worried) regarding their risk of neck/back pain flying fast jets, and, the risk that neck/back pain may one day preclude them from flying fast jets.

Height, body mass, and neck circumference were measured using previously published methods (Norton and Olds 1996). Cervical spine range of motion (ROM) (flexion, extension, rotation) was measured using a CROM Device (Performance Attainment Associates, USA), and trunk rotation ROM was measured using a bubble inclinometer (Baseline, USA), using previously published methods (Fletcher and Bandy 2008; Iveson et al. 2010). Isometric maximal strength of the neck and trunk was measured using David Spine Devices (David Health, Finland) for flexion, extension, rotation, and lateral flexion for both regions. Predicted one-repetition-maximum (1RM) strength testing was conducted for the squat, dead-lift, bench-press, and pull-up (Baechle et al. 2000). Cardiorespiratory testing was conducted on a Concept2 Model-D rowing ergometer (Concept2, USA) for maximal performance on 500 m row and 5-minute row. Further detail on baseline data collection methods is provided in Online Resource 1.

#### Weekly data collection

Flying external workloads were quantified by *weekly number of flights* and *weekly total flight time* (hours) of flights per week (Monday-Sunday), with data retrieved from the flight resource and scheduling system where FJA logged data following each flight. Flying internal workloads were captured for each flight using session rating of perceived exertion (sRPE) (0–10 scale) for two differential measures regarding physical demand and neck demand, and multiplied by flight duration (minutes) to provide a workload score in arbitrary units (AU). Weekly scores for each were tallied providing *acute flying neck workload*, and *acute flying physical workload*, and tallied across a four-week period to provide *chronic flying neck workload*, and *chronic flying physical workload*. S&C internal workloads were similarly calculated for *acute S&C physical workload* and *acute S&C neck workload*, *chronic S&C physical workload* and *chronic S&C neck workload*. FJA entered sRPE and session duration following each FJA S&C sessionand for their own self-directed sessions using a custom S&C log in Smartabase (Fusion Sport, Australia). Combined internal workload scores were also created by summating the respective flying and S&C internal workloads: *acute combined physical workload*, *acute combined neck workload*, *chronic combined physical workload*, and *chronic combined neck workload*. Further detail on workload measures is provided in Online Resource 1.

Psychosocial stress-recovery (well-being) was rated on a 0–6 scale comprising four subscales (using 10 questions) from the Recovery-Stress Questionnaire – Sport (RESTQ-S-52) (Kellmann and Kallus 2001) including: fatigue, general wellbeing, sleep quality, and fitness/injury. FJA completed the questions once per week using a custom log using Smartabase (Fusion Sport, Australia). An acute and chronic score for each was based upon the scores in a given week, and the average of acute scores over a four-week period respectively. Further detail on well-being measures is provided in Online Resource 1.

At the time of this study, RAAF had two FJA-specific S&C programs in which FJA were encouraged to participate: a gym-based program (free-weight strength exercises and aerobic/anaerobic conditioning on rowing ergometer and assault bike), and a targeted neck and back strengthening program using David equipment (David Health, Finland) (strength exercises using pin-loaded machines targeting each plane of movement of the neck and trunk). Gym program participation was captured by S&C staff present and via FJA logging sessions in the S&C log using Smartabase, and David program participation was captured by software within the David equipment. Weekly participation was considered to have occurred when FJA completed two sessions (on separate days) of a program in a week. Further details on S&C programs are provided in Online Resource 1.

### Statistical analysis

To ensure exposures (e.g. flying workloads) preceded the outcome of interest (a new NRMCE), exposures in a given week were evaluated against the occurrence of a new NRMCE recorded the following week, with this lag period (one week) set by the frequency of the UCFJAMQ’s use. This was used for both weekly and baseline measures as adjusted models included confounders that were measured weekly (e.g. flying workloads).

Mixed-effects logistic regression was undertaken using lme4 package in R (4.4.1). R-packages used in this study are listed in Online Resource 1. Two model types were evaluated: a ‘*simple model*’ and an ‘*adjusted model*’. Simple models adjusted only for ‘previous experience of an NRMCE in the current reporting period’ (in an effort to reduce the bias of an FJA having recently experienced, or currently experiencing, an NRMCE). Adjusted models adjusted for previous experience of an NRMCE in current reporting period and additional potential confounders based upon current literature and clinical reasoning, including: age, previous neck related complaint in the past 12 months, gender, recent flying exposure, and recent S&C exposure (adjustors used for each analysis are listed in results).

To account for the potential dependency with (a) repeated data from the same individuals, and (b) unique flying programs, airframe, and aircrew of each unit within each reporting period, the suitability of random effects (i.e. multi-level) modelling was examined. An empty fixed effects model (logistic regression) was compared against three random effects model specifications with random intercepts for: (a) individual FJA (ID), (b) individual unit for a given reporting period (UnitRP), and (c) a cross-nested random effects with both included. Akaike information criterion (AIC) from each model was compared followed by model likelihood ratio test (MLRT) to confirm any AIC reductions and thus justify the inclusion of a given random effect model specification (Schweinberger 2024).

Variables with potential to vary within individual FJA were assessed with a random slope, with comparison made to a random intercept only model made using AIC followed by MLRT to confirm any AIC reductions and thus justify the inclusion of a random slope (Winter 2019). Continuous variables were assessed for non-linearity using restricted cubic splines (RCS) using three, four and five knots placed at their respective quantile mark points (Harrel 2001), and again assessed using AIC followed by MLRT (Harrel 2001; Rhon et al. 2022).

Convergence issues were addressed by scaling continuous predictors (by 10, 100, or 1000 where appropriate), substituting the optimisers (provided in Online Resource 1), and reconsidering the inclusion of any random slopes (Winter 2019). Independence of variables (exposure of interest and confounders being adjusted for) was assessed by evaluating correlation matrices of model outputs, and calculating variance inflation factors (VIF), with a cutoff VIF value of > 3 (Zuur et al. 2010).

All data were assessed for missingness prior to analyses. Where baseline data for previous neck pain (at all, previous 12 months), total flying fast jet hours, and height was missing for a given reporting period, logic was performed where possible using an individual’s data from the previous reporting period. Missing sRPE entries were imputed using multivariate imputation by chained equations (MICE) with predicted mean matching (using MICE package) as has previously demonstrated to be an accurate method with missing sRPE data rates of 50% missing (Bache-Mathiesen et al. 2022). Number of imputations was set as the percentage of missing data (i.e. if 20% was missing, imputations = 20) (Austin et al. 2021), and iterations set as 10. Imputation model for sRPE included the variables: individual FJA (ID); age; gender; flying role; cockpit role; relevant duration (flight, S&C session); a combined variable specific to reporting period, unit and week; relevant physical sRPE (flight, S&C session); relevant neck sRPE (flight, S&C session); and, the outcome variable (new episode of NRMC the following week). Convergence and density plots were visually inspected. Given the complexity of undertaking MICE across multiple variables, using sRPE to then create workload scores which were then summated for given period, and then using these workload values within a large number of multivariable analyses, we computed an average of the plausible values provided by MICE (Ren et al. 2023) before calculating workload scores (i.e. multiplying sRPE by duration). Available case analysis was undertaken for the remaining variables in order to retain as much available data from each case as possible (Ren et al. 2023).

## Results

A total of 279 FJA were included (following removal of FJA who were: present for less than half a reporting period; not present the following week and/or had not completed the UCFJAMQ). A mean of 218 (range 209–224) FJA were included in each 5-month reporting period. Baseline data relating to the 279 FJA is provided in Table 1, with missing data ranging from 0% for demographic data to 77% for gym testing. Missing data for sRPE entries were 33% for flights, 19% for David sessions, and 21% for Gym sessions, which reduced to 0% following MICE. Wellbeing entries had 35% missing, and UCFJAMQ had 37% missing. There were 320 new NRMCEs included, with a median duration of 1 week (interquartile range 1–2 weeks), mean weekly prevalence of 4.1% (SD 2.3), and mean 5-month reporting period prevalence of 22.4% (range 15.3–28.5%).

**Table 1:** Participant baseline data

Item	Category Count (%)	Mean (SD)	Missing data
Demographic			
Age (years)		32.9 (7.92)	9%
Gender - Female	15 (5%)		0%
Cockpit role			1%
Pilot	*Not provided* ^b^		
ACO (WSO or EWO)	*Not provided* ^b^		
Flying role ^a^			0%
Student	*Not provided* ^b^		
Operational	*Not provided* ^b^		
Instructor	*Not provided* ^b^		
Airframe ^a^			0%
F-18 F	223 (26%)		
EA-18G	112 (13%)		
F-35	84 (10%)		
F/A-18	205 (24%)		
Hawk-127	246 (28%)		
Questionnaire			
Total fighter aircraft hours ^a^		1240 (1301)	13%
Previous neck pain ^a^			
In past 3 months - Yes	50 (11%)		46%
In past 12 months - Yes	131 (26%)		43%
In past at all - Yes	320 (53%)		31%
Worry regarding future spinal pain^a^			
Risk of flying fighter aircraft		3.3 (2.2)	47%
May preclude from flying fighter aircraft		3.2 (2.5)	47%
Anthropometry			
Height (cm)		180.1 (6.0)	10%
Body mass (kg) ^a^		81.5 (10.4)	35%
Neck circumference (cm) ^a^		38.2 (2.5)	42%
Range of Motion ^a^			
Neck flexion (deg)		58.3 (9.0)	42%
Neck extension (deg)		74.9 (10.0)	42%
Neck transverse plane total (deg)		138.5 (15.3)	42%
Trunk transverse plane total (deg)		114.6 (21.8)	42%
Spinal Isometric Strength – Absolute ^a^			
Neck flexion (kg)		30.6 (10.4)	40%
Neck extension (kg)		59.9 (14.5)	40%
Neck rotation mean (kg)		15.8 (4.9)	40%
Neck lateral flexion mean (kg)		45.1 (12.3)	40%
Trunk flexion (kg)		204.8 (59.8)	40%
Trunk extension (kg)		299.8 (82.0)	40%
Trunk rotation mean (kg)		195.7 (55.0)	40%
Trunk lateral flexion mean (kg)		207.5 (60.2)	40%
Spinal Isometric Strength – Relative ^a^			
Neck flexion (kg per kg BM)		0.38 (0.12)	40%
Neck extension (kg per kg BM)		0.72 (0.16)	40%
Neck rotation (kg per kg BM)		0.19 (0.06)	40%
Neck lateral flexion (kg per kg BM)		0.56 (0.14)	40%
Trunk flexion (kg per kg BM)		2.52 (0.62)	40%
Trunk extension (kg per kg BM)		3.68 (0.88)	40%
Trunk rotation (kg per kg BM)		2.40 (0.58)	40%
Trunk lateral flexion (kg per kg BM)		2.54 (0.64)	40%
Gym testing – Absolute ^a^			
Pull-up predicted 1RM (kg)		104.3 (16.8)	77%
Bench press predicted 1RM (kg)		85.6 (20.2)	73%
Deadlift predicted 1RM (kg)		132.3 (32.5)	76%
Squat predicted 1RM (kg)		106.4 (24.0)	74%
500 m row time (seconds)		95.6 (7.2)	76%
5-minute row (m)		1336 (84.4)	77%
Gym testing – Relative ^a^			
Pull-up (kg per kg BM)		1.28 (0.15)	77%
Bench press (kg per kg BM)		1.04 (0.21)	73%
Deadlift (kg per kg BM)		1.62 (0.35)	76%
Squat (kg per kg BM)		1.30 (0.26)	74%

^a^Average or tallied across reporting periods for the purpose of this table

^b^Data not provided due to security reasons

ACO, air combat officer; WSO, weapons systems officer; EWO, electronic warfare officer; BM, body mass; deg, degrees; cm, centimetres; kg, kilograms; 1RM, one repetition maximum; m, metres

Details of the 44 baseline and 26 weekly factors investigated in the adjusted models are displayed in Tables 2 and 3 respectively, along with their model specifications and findings. Details of the simple models can be found in Online Resource 2. Eight of the simple and eight of the adjusted models were best fitted with non-linear models. Significant relationships were found in 31 of the simple models and 22 of the adjusted models.

**Table 2:** Findings for baseline data collection—Adjusted models

Factor	Model specifications	Additional adjustors	Observations (n FJA)	New NRMC episodes	Observation mean (SD), or # per category	OR (95% CI) ^b^	*p*
Demographics							
Age (per year)	IO (2RI)	Gender, neck pain past 12mo, acute fly physical WL	6643 (222)	164	32.2 (8.1)	1.00 (0.97–1.03)	0.866
Gender:	IO (2RI)	Age, neck pain past 12mo, acute fly physical WL	6698 (223)	164			
Male					493 (6205)	1.00 ^c^	
Female						1.10 (0.51–2.36)	0.812
Flying role:	IO (2RI)	Age, gender, neck pain past 12mo, acute fly physical WL	6698 (223)	164			
Student					1265	1.00 ^c^	
Front Line					3814	1.11 (0.56–2.20)	0.758
Instructor					1619	1.56 (0.70–3.47)	0.278
Cockpit role:	IO (2RI)	Age, gender, neck pain past 12mo, acute fly physical WL	6698 (223)	164			
Pilot					5300	1.00 ^c^	
Back seater					1398	0.8 (0.46–1.39)	0.426
Current aircraft:	IO (2RI)	Age, gender, neck pain past 12mo, acute fly physical WL	6698 (223)	164			
F/18F					1633	1.00 ^c^	
EA-18G					969	1.00 (0.43–2.34)	0.998
F-35					274	1.61 (0.59–4.38)	0.354
F/A-18 C					1304	0.7 (0.31–1.56)	0.384
Hawk					2518	0.91 (0.48–1.75)	0.787
Questionnaire							
Neck pain - past 3mo:	IO (1RI)	Age, gender, acute fly physical WL	6307 (221)	156			
No					5590	1.00 ^c^	
Yes					717	2.58 (1.56–4.27)	< 0.001*
Neck pain - past 12mo:	IO (2RI)	Age, gender, acute fly physical WL	6698 (223)	164			
No					5018	1.00 ^c^	
Yes					1680	2.63 (1.72–4.02)	< 0.001*
Neck pain - past at all:	IO (2RI)	Age, gender, acute fly physical WL	6698 (223)	164			
No					3741	1.00 ^c^	
Yes					2957	2.54 (1.63–3.97)	< 0.001*
Total fighter hours (per 100 h)	IO (2RI)	Gender, neck pain past 12mo, acute fly physical WL	6698 (223)	164	11.94 (14.52)	1.00 (0.98–1.02)	0.936
Worry - Risk of neck/back pain flying fighters (per 1 unit)	IO (1RI)	Age, gender, neck pain past 12mo, acute fly physical WL	6213 (221)	155	3.17 (2.19)	1.15 (1.05 –1.25)	0.002 *
Worry - Risk neck/back pain may preclude you from fighters in future (per 1 unit)	IO (1RI)	Age, gender, neck pain past 12mo, acute fly physical WL	6213 (221)	155	3.15 (2.47)	1.13 (1.04–1.22)	0.003 *
Anthropometry							
Height (per 10 cm)	IO (1RI)	Age, gender, neck pain past 12mo, acute fly physical WL	6119 (216)	154	17.99 (0.61)	0.89 (0.58–1.35)	0.583
Body mass (per 10 kg)	IO (1RI)	Age, gender, neck pain past 12mo, acute fly physical WL	6152 (218)	154	8.07 (1.05)	0.86 (0.67–1.10)	0.226
Neck circumference (per cm)	IO (1RI)	Age, gender, neck pain past 12mo, acute fly physical WL	5794 (207)	149	38.03 (2.59)	0.91 (0.81–1.02)	0.104
Range of motion							
Neck flexion (per 10°)	IO (1RI)	Age, gender, neck pain past 12mo, acute fly neck WL	5307 (203)	133	5.81 (0.88)	0.87 (0.68–1.11)	0.257
Neck extension (per 10°)	IO (1RI)	Age, gender, neck pain past 12mo, acute fly neck WL	5307 (203)	133	7.55 (1.02)	0.88 (0.71–1.10)	0.271
Neck transverse plane total (per 10°)	IO (1RI)	Age, gender, neck pain past 12mo, acute fly neck WL	5850 (205)	149	13.86 (1.55)	0.92 (0.80–1.05)	0.225
Trunk transverse plane total (per 10°)	IO (1RI)	Age, gender, neck pain past 12mo, acute fly neck WL	5823 (206)	151	11.40 (2.15)	1.07 (0.98–1.18)	0.149
Isometric spinal strength – Absolute							
Neck flexion (per 10 kg)	IO (1RI)	Age, gender, neck pain past 12mo, acute fly neck WL	5733 (213)	148	3.11 (1.06)	0.88 (0.72–1.07)	0.188
Neck extension (per 10 kg)	IO (1RI)	Age, gender, neck pain past 12mo, acute fly neck WL	5768 (214)	148	5.74 (1.49)	0.98 (0.84–1.14)	0.801
Neck rotation (per 10 kg)	RCS 3 (1RI)	Age, gender, neck pain past 12mo, acute fly neck WL	5660 (212)	148	1.59 (0.5)	0.36 (0.14–0.94)	0.036 *
						3.14 (1.12–8.86)	0.03 *
Neck lateral flexion (per 10 kg)	IO (1RI)	Age, gender, neck pain past 12mo, acute fly neck WL	5758 (214)	148	4.55 (1.28)	0.94 (0.80–1.10)	0.439
Trunk flexion (per 10 kg)	RCS 3 (1RI)	Age, gender, neck pain past 12mo, acute fly neck WL	5758 (214)	147	19.6 (5.45)	0.9 (0.82-1.00)	0.043 *
						1.14 (1.02–1.27)	0.025 *
Trunk extension (per 10 kg)	IO (1RI)	Age, gender, neck pain past 12mo, acute fly neck WL	5731 (213)	141	29.59 (8.47)	0.98 (0.96–1.01)	0.21
Trunk rotation (per 10 kg)	IO (1RI)	Age, gender, neck pain past 12mo, acute fly neck WL	5749 (214)	147	19.51 (5.64)	0.99 (0.95–1.02)	0.485
Trunk lateral flexion (per 10 kg)	IO (1RI)	Age, gender, neck pain past 12mo, acute fly neck WL	5778 (214)	147	20.56 (6.27)	0.99 (0.96–1.03)	0.602
Isometric spinal strength – Relative							
Neck flexion (kg per kg BM)	IO (1RI)	Age, gender, neck pain past 12mo, acute fly neck WL	5722 (212)	148	0.39 (0.12)	0.33 (0.06–1.80)	0.201
Neck extension (kg per kg BM)	IO (1RI)	Age, gender, neck pain past 12mo, acute fly neck WL	5757 (213)	148	0.71 (0.17)	1.07 (0.29–3.98)	0.919
Neck rotation (kg per kg BM)	IO (1RI)	Age, gender, neck pain past 12mo, acute fly neck WL	5649 (211)	148	0.2 (0.06)	0.5 (0.01–19.63)	0.708
Neck lateral flexion (kg per kg BM)	IO (1RI)	Age, gender, neck pain past 12mo, acute fly neck WL	5747 (213)	148	0.56 (0.15)	0.65 (0.16–2.58)	0.541
Trunk flexion (kg per kg BM)	I&S (1RI)	Age, gender, neck pain past 12mo, acute fly neck WL	5747 (213)	147	2.42 (0.55)	0.87 (0.48–1.58)	0.642
Trunk extension (kg per kg BM)	RCS 3 (1RI)	Age, gender, neck pain past 12mo, acute fly neck WL	5720 (212)	141	3.67 (0.92)	1.33 (0.82–2.14)	0.249
						0.57 (0.32–1.01)	0.052
Trunk rotation (kg per kg BM)	IO (1RI)	Age, gender, neck pain past 12mo, acute fly neck WL	5738 (213)	147	2.41 (0.6)	0.92 (0.66–1.28)	0.606
Trunk lateral flexion (kg per kg BM)	IO (1RI)	Age, gender, neck pain past 12mo, acute fly neck WL	5767 (213)	147	2.54 (0.67)	0.95 (0.70–1.29)	0.723
Gym-based testing – Absolute							
Pull-up (per 10 kg)	IO (1RI)	Age, gender, neck pain past 12mo, acute fly neck WL	2114 (91)	39	10.08 (1.68)	Model failed ^d^	
Bench press (per 10 kg)	IO (1RI)	Age, gender, neck pain past 12mo, acute fly neck WL	2366 (106)	45	8.28 (1.86)	Model failed ^d^	
Deadlift (per 10 kg)	IO (1RI)	Age, gender, neck pain past 12mo, acute fly neck WL	2257 (99)	42	12.65 (2.76)	Model failed ^d^	
Squat (per 10 kg)	IO (1RI)	Age, gender, neck pain past 12mo, acute fly neck WL	2352 (104)	45	10.39 (2.17)	Model failed ^d^	
5 min row (per 100 m)	IO (1RI)	Age, gender, neck pain past 12mo, acute fly neck WL	2110 (94)	35	13.29 (0.86)	Model failed ^d^	
500 m row (per 10 s)	IO (1RI)	Age, gender, neck pain past 12mo, acute fly neck WL	2159 (95)	36	9.69 (0.78)	Model failed ^d^	
Gym-based testing – Relative							
Pull-up (kg per kg BM)	IO (1RI)	Age, gender, neck pain past 12mo, acute fly neck WL	2114 (91)	39	1.26 (0.14)	Model failed ^d^	
Bench press (kg per kg BM)	IO (1RI)	Age, gender, neck pain past 12mo, acute fly neck WL	2366 (106)	45	1.02 (0.19)	Model failed ^d^	
Deadlift (kg per kg BM)	IO (1RI)	Age, gender, neck pain past 12mo, acute fly neck WL	2257 (99)	42	1.57 (0.3)	Model failed ^d^	
Squat (kg per kg BM)	IO (1RI)	Age, gender, neck pain past 12mo, acute fly neck WL	2352 (104)	45	1.29 (0.23)	Model failed ^d^	

*p < 0.05

^b^For categorical data, Odds Ratio provided for given category compared to reference category; For continuous data, Odds Ratio provided per unit increase as noted in the factor column.

^c^Reference category.

^d^Model failed due to boundary singular error indicating overfitting secondary to low numbers of FJA undertaking such testing.

1RI, one random intercept only (individual FJA); 2RI, two random intercepts (cross-nested: individual FJA and squadron for given semester); 3mo, 3 months; 12mo, 12 months; BW, body mass; IO, random intercept only; I&S, random intercept and random slope; OR, odds ratio; RCS-3, restricted cubic splines using three knots; RCS-4, restricted cubic splines using four knots; SD, standard deviation; NRMC, neck related musculoskeletal complaint; WL, workload

**Table 3:** Findings for weekly data collection—Adjusted models

Factor	Model specifications	Additional adjustors	Observations (*n* FJA)	New NRMC episodes	Observation mean (SD)	OR (95% CI) ^b^	*p*
External Flying Workload							
Weekly number of flights (per 1 flight)	IO (2RI)	Age, gender, neck pain past 12mo, acute fly neck WL	6698 (223)	164	*Not provided* ^a^	1.13 (1.02–1.24)	0.014 *
Weekly total flight time (per 1 h)	IO (2RI)	Age, gender, neck pain past 12mo, acute fly neck WL	6698 (223)	164	*Not provided* ^a^	1.04 (0.98–1.11)	0.208
Internal Flying Workload							
Acute flying neck workload (per 1000 units)	IO (2RI)	Age, gender, neck pain past 12mo, acute S&C neck WL	6698 (223)	164	0.37 (0.43)	1.59 (1.19–2.12)	0.002 *
Acute flying physical workload (per 1000 units)	IO (2RI)	Age, gender, neck pain past 12mo, acute S&C physical WL	6698 (223)	164	0.45 (0.5)	1.48 (1.13–1.93)	0.004 *
Chronic flying neck workload (per 1000 units)	RCS-3 (2RI)	Age, gender, neck pain past 12mo, chronic S&C neck WL	6698 (223)	164	1.45 (1.2)	2.08 (1.31–3.30)	0.002 *
						0.55 (0.35–0.86)	0.009 *
Chronic flying physical workload (per 1000 units)	RCS-3 (2RI)	Age, gender, neck pain past 12mo, chronic S&C physical WL	6698 (223)	164	1.76 (1.42)	1.82 (1.22–2.71)	0.003 *
						0.62 (0.42–0.92)	0.016 *
Internal S&C Workload							
Acute S&C neck workload (per 1000 units)	IO (2RI)	Age, gender, neck pain past 12mo, acute fly neck WL	6698 (223)	164	0.29 (0.34)	1.8 (1.18–2.76)	0.007 *
Acute S&C physical workload (per 1000 units)	IO (2RI)	Age, gender, neck pain past 12mo, acute fly physical WL	6698 (223)	164	0.59 (0.64)	1.47 (1.16–1.86)	0.002 *
Chronic S&C neck workload (per 1000 units)	IO (2RI)	Age, gender, neck pain past 12mo, chronic fly neck WL	6698 (223)	164	1.14 (1.17)	1.2 (1.06–1.37)	0.005 *
Chronic S&C physical workload (per 1000 units)	IO (2RI)	Age, gender, neck pain past 12mo, chronic fly physical WL	6698 (223)	164	2.33 (2.17)	1.1 (1.02–1.19)	0.016 *
Internal combined workload							
Acute combined neck workload (per 1000 units)	IO (2RI)	Age, gender, neck pain past 12mo	6698 (223)	164	0.66 (0.58)	1.65 (1.31–2.09)	< 0.001 *
Acute combined physical workload (per 1000 units)	IO (2RI)	Age, gender, neck pain past 12mo	6198 (223)	164	1.04 (0.85)	1.47 (1.23–1.76)	< 0.001 *
Chronic combined neck workload (per 1000 units)	RCS-3 (2RI)	Age, gender, neck pain past 12mo	6698 (223)	164	2.59 (1.83)	1.73 (1.25–2.39)	0.001 *
						0.64 (0.44–0.91)	0.015 *
Chronic combined physical workload (per 1000 units)	IO (2RI)	Age, gender, neck pain past 12mo	6698 (223)	164	4.09 (2.79)	1.11 (1.05–1.18)	< 0.001 *
S&C Participation							
David 8-week participation (per week) ^c d^	IO (1RI)	Age, gender, neck pain past 12mo, acute fly physical WL, FF Gym 8-week participation	5302 (223)	121	1.95 (2.36)	1.04 (0.95–1.14)	0.37
David 12-week participation (per week) ^c d^	IO (1RI)	Age, gender, neck pain past 12mo, acute fly physical WL, FF Gym 12-week participation	3909 (219)	81	2.78 (3.19)	0.96 (0.89–1.05)	0.388
FF Gym 8-week participation (per week) ^c d^	IO (1RI)	Age, gender, neck pain past 12mo, acute fly physical WL, David 8-week participation	5302 (223)	121	2.94 (2.74)	0.96 (0.89–1.04)	0.332
FF Gym 12-week participation (per week) ^c d^	IO (1RI)	Age, gender, neck pain past 12mo, acute fly physical WL, David 12-week participation	3909 (219)	81	4.3 (3.84)	0.98 (0.92–1.05)	0.561
Wellbeing							
Acute fatigue (per 1 unit)	IO (1RI)	Age, gender, neck pain past 12mo, acute fly neck WL	5969 (220)	144	1.06 (1.06)	1.1 (0.93–1.31)	0.246
Chronic fatigue (per 1 unit)	IO (1RI)	Age, gender, neck pain past 12mo, chronic fly neck WL	5503 (218)	128	1.07 (0.91)	1.24 (0.99–1.56)	0.062
Acute general wellbeing (per 1 unit)	IO (1RI)	Age, gender, neck pain past 12mo, acute fly neck WL	5962 (220)	143	1.67 (1.2)	0.99 (0.83–1.17)	0.873
Chronic general wellbeing (per 1unit)	IO (1RI)	Age, gender, neck pain past 12mo, chronic fly neck WL	5488 (218)	127	1.68 (1.11)	1.15 (0.95–1.40)	0.147
Acute sleep quality (per 1 unit)	RCS-3 (1RI)	Age, gender, neck pain past 12mo, acute fly neck WL	5962 (220)	144	1.88 (1.19)	1.55 (1.03–2.34)	0.035 *
						0.63 (0.40–0.99)	0.047 *
Chronic sleep quality (per 1 unit)	RCS-3 (1RI)	Age, gender, neck pain past 12mo, chronic fly neck WL	5490 (218)	128	1.9 (1.05)	2.29 (1.25–4.19)	0.007 *
						0.51 (0.28–0.95)	0.033 *
Acute fitness/injury (per 1 unit)	I&S (1RI)	Age, gender, neck pain past 12mo, acute fly neck WL	6044 (221)	146	2.05 (0.61)	0.78 (0.47–1.29)	0.33
Chronic fitness/injury (per 1 unit)	IO (1RI)	Age, gender, neck pain past 12mo, chronic fly neck WL	5575 (219)	131	2.05 (0.5)	1.33 (0.90–1.97)	0.15

*p < 0.05

^a^Not provided for security reasons.

^b^Odds Ratio provided per unit increase as noted in the factor column.

^c^A week of participation indicates one week where two sessions of a given program were undertaken (e.g. a participation value of 8 indicates an individual has performed a given program twice per week on 8 separate weeks).

^d^Individuals had to be present in the previous 7 weeks, or previous 11 weeks, AND the current week for their data to be included in these analyses.

1RI, one random intercept only (individual FJA); 2RI, two random intercepts (cross-nested: individual FJA and squadron for given semester); 12mo, 12 months; IO, random intercept only; I&S, random intercept and random slope; OR, odds ratio; RCS-3, restricted cubic splines using three knots; RCS-4, restricted cubic splines using four knots; SD, standard deviation; NRMC, neck related musculoskeletal complaint.

Significant findings in adjusted models of baseline data included: previous neck pain (in past 3-months, past 12-months, and at all) (Odds Ratio [OR]: 2.58 (95%CI 1.56–4.27), 2.63 (1.72–4.02), 2.54 (1.63–3.97) respectively); and higher scores on the two worry ratings (OR 1.15 (1.05–1.25) and 1.13 (1.04–1.22) per 1 unit). Significant non-linear relationships with risk of new NRMCE were found for isometric neck rotation and trunk flexion absolute strength where values up to 15.6 kg and 190 kg had OR’s of 0.36 (0.14–0.94) and 0.9 (0.82-1.00) respectively, and values above these had OR’s of 3.14 (1.12–8.86) and 1.14 (1.02–1.27) respectively. All gym-based measures in the adjusted models failed due to overfitting due to low numbers of FJA undertaking such testing.

Significant findings in adjusted weekly data included: number of flights (OR 1.13 (1.02–1.24) per flight); acute flying neck and physical workload (OR 1.59 (1.19–2.12) and 1.48 (1.13–1.93) per 1000 unit increase respectively); acute S&C neck and physical workload (OR 1.8 (1.18–2.76) and 1.47 (1.16–1.86) per 1000 unit increase respectively); chronic S&C neck and physical workload (OR 1.2 (1.06–1.37) and 1.1 (1.02–1.19) per 1000 unit increase respectively); acute combined neck and physical workload, and chronic combined physical workload (OR 1.65 (1.31–2.09), 1.47 (1.23–1.76), and 1.11 (1.05–1.18) per 1000 unit increase respectively. Significant non-linear relationships with risk of new NRMCE were found for chronic flying neck and physical workload, and chronic combined neck workload, where values up to 1236, 1512, and 2227 AU had OR’s of 2.08 (1.31–3.3), 1.82 (1.22–2.71), and 1.73 (1.25–2.39) per 1000 unit increase respectively, while values above these had OR’s of 0.55 (0.35–0.86), 0.62 (0.42–0.92), and 0.64 (0.44–0.91) respectively. Acute and chronic sleep quality also demonstrated significant non-linear relationships where scores up to 2.0 and 1.9 had OR’s of 1.55 (1.03–2.34) and 2.29 (1.25–4.19) respectively, while values above these had OR’s of 0.63 (0.40–0.99) and 0.51 (0.28–0.95) respectively.

As per other FJA publications (Honkanen et al. 2018) some means and counts pertaining to personnel and flying rates are not provided in for security reasons.

## Discussion

This is the first study to prospectively examine potential contributory risk factors of NRMCEs among FJA across a wide range of demographic, physical capacity, recovery-stress states, and workload measures. Of the 70 factors investigated, significant relationships were found in 22 of the adjusted models. The majority of effect sizes (and potential effect sizes for continuous variables) are considered small (OR of 1.5 or 0.67) to medium (OR of 2.5 or 0.40) (Rosenthal 1996), reinforcing the multifactorial nature of NRMCEs and the importance of multifaceted prevention. Our findings as to which factors are, and are not, related to risk of NRMCE are critical to improving the effectiveness and efficiency of prevention programs.

Self-reports of previous neck pain demonstrated significant relationships with weekly risk of a new NRMCE (OR’s 2.54–2.63), which is consistent with previous research regarding neck pain (Kim et al. 2018) and injuries in military populations (Rhon et al. 2021). Self-reported worry measures were among the few other baseline measures significantly related to weekly risk of new NRMCE. While not previously considered in FJA, previous work among athletes returning from injury observed that those with higher ‘re-injury worry’ had a higher re-injury risk (Christakou et al. 2022). Our results build upon this, highlighting that risks of worry are not isolated to just those returning to an activity post-injury, nor just sporting populations. While previous neck pain is not a modifiable risk factor, self-reported worry likely is, and consideration of ways to reduce such worry is warranted.

Higher acute internal flying workloads (where duration and intensity using sRPE are incorporated) demonstrated significant linear relationships with weekly risk of a new NRMCE, whereas no such relationship was seen with weekly flying duration. This highlights the importance of exposure measures also capturing intensity, not just duration, when evaluating NRMCE risk in FJA. Interestingly, chronic internal flying workloads demonstrated significant non-linear relationships, whereby neck and physical workloads up to 1240 AU and 1510 AU respectively increased risk, but higher loads reduced such risk. While we did not evaluate relative workloads (such as acute to chronic workload ratios) due to recent criticisms (Wang et al. 2020), these findings suggest there may indeed be benefit in maintaining consistent higher workloads as advocated by relative-workload models (Windt and Gabbett 2017). Close attention should therefore be given to internal flying workloads of FJA both during and leading up to demanding flying programs (such as air-to-air programs where workloads are typically high), and the programming of such demanding flying programs should be avoided following periods of low flying workload. Integration of sRPE into existing flight systems (as opposed to standalone systems used in this study) where FJA immediately log data following each flight would also address the issues with missing data.

Cervical spine rotation strength (absolute) was the only cervical isometric strength measure demonstrating a significant relationship with weekly risk of a new NRMCE. Capacities up to 15.6 kg reduced the risk with a medium effect size, but greater capacities increased risk with medium to large effect sizes. A similar relationship was found for trunk flexion strength (absolute), where capacities up to 190 kg reduced the risk, but greater capacities increased risk. This suggests increasing cervical rotation and/or trunk flexion strength of weaker FJA may be beneficial, but a ceiling effect is present whereby seeking capacities beyond this ceiling may indeed be harmful. Interestingly, none of the strength measures adjusted for body weight (i.e. relative strength) demonstrated significant relationships with weekly risk of a new NRMCE, which may reflect + Gz forces experienced by FJA in the jet are fixed, the mass of helmet mounted equipment is fixed, and combined helmet and head mass across body different body masses has lower variation than does body mass.

The gym-based measures (predicted 1RM testing, rowing ergometer testing) failed with adjusted models due to too few FJA participating. However, the simple models demonstrated no association with weekly risk of new NRMCE, contrasting previous research where higher cardiorespiratory fitness had a protective relationship with injury risk among military populations (Lovalekar et al. 2021). Given the relationship of rowing performance with limb lengths and skill/technique (Choszcz et al. 2012), it may be that rowing ergometer testing was not an accurate measure of cardiorespiratory fitness in FJA. However, our results do support those of our previous systematic review where cardiorespiratory fitness was not associated with neck pain in FJA (Wallace et al. 2021). Seeking improvements in these gym-based measures, and most of the spinal isometric strength measures, therefore appear to have no value in reducing the risk of NRMCE in FJA.

Given the content of the FJA-specific gym and David S&C programs, it is unsurprising that higher participation in either of program over eight and twelve week periods had no effect on weekly risk of a new NRMCE. Recent work has demonstrated, that unlike sporting populations, S&C programs in military populations have had weak to no effect on reducing injury rates (Bunn et al. 2024; Tingelstad et al. 2024). These authors suggested military-focussed programs often contribute to increased workloads placed upon participants which inherently increases their injury risk. This is supported by our findings whereby all acute and chronic S&C workload measures indeed linearly increased weekly risk of new NRMCE. We therefore agree with previous recommendations that S&C prevention programs in military populations should seek to keep workloads low via short durations (such as ‘warm-up’ programs), and be targeted to demonstrated risk factors (Bunn et al. 2024; Tingelstad et al. 2024).

Of the wellbeing measures, only acute and chronic sleep quality measures demonstrated significant relationships with weekly risk of new NRMCE. Interestingly, both acute and chronic measures of sleep quality had nonlinear relationships with scores up to 2.0 and 1.9 respectively relating to increased risk, while higher scores related to reduced risk. Previous research has found similar contrasting relationships with injury risk and fatigue scores (Jones et al. 2017), and suggested such relationships may be explained by players who recognised higher fatigue levels reduced their playing/training intensity and vice versa. Monitoring and efforts to improve sleep quality therefore appear worthy targets to reduce risk of NRMCE, and potentially reduce performance restrictions that FJA may self-impose.

### Strengths and limitations

The strengths of our study are embedded in the methodology. This study was prospective, included a relatively large sample size reflecting a large proportion of RAAF FJA, and was conducted over four 5-month surveillance periods, demonstrating the ‘real world’ nature of our findings. We used the UC-FJAMQ, a validated surveillance tool which was developed upon expert-consensus, and we used a lag period to ensure exposure to potential risk factors and confounders preceded the outcome. Statistical analyses included Mixed-effects modelling to account for dependency within the data, and assessment was undertaken of non-linear relationships. Potential confounders were addressed, with selection informed by clinical reasoning and research beyond FJA literature alone. These methods are congruent with previous recommendations (Losciale et al. 2023), and strengthen the confidence in our findings regarding contributory risk factors for neck pain among FJA.

Our study however has some limitations. Missing data reduced data available for analysis, and hampered evaluation of adjusted models for gym-based measures. Future work should seek to: improve data entry of FJA; investigate barriers of FJA undertaking baseline testing; and, consider the ability for FJA and/or embedded healthcare providers to report FJA short leave periods to thus justify missed data entry. Secondly, MICE was used only for missing sRPE data as opposed to all missing data. MICE has demonstrated accuracy with imputing sRPE data (Bache-Mathiesen et al. 2022), but we were unsure of its accuracy with our other data. Future work should evaluate the accuracy of MICE with physical testing data and wellbeing measures. Thirdly, internal workload data used weekly blocks as opposed to 7-day rolling averages as has been recommended. Given the outcome (NRMCE) was collected on a weekly (as opposed daily) basis, 7-day rolling averages would have been hampered by the incongruent collection of NRMCE, and our previous experience with FJA is that daily UC-FJAMQ entries are highly unpalatable. Fourthly, we did not conduct correction procedures (e.g. Bonferroni) to address the potential for Type I error given the number of analyses we conducted. However, our analytical approach differs in several important respects that make such procedures unnecessary and their use would be overly conservative in this context (i.e. they would incur a greater risk of Type II error). For instance, the potential risk factors we analysed were pre-specified based on current literature and clinical reasoning, and were not the product of a broad, exploratory analysis. Furthermore, mixed-effects logistic regression permitted the modelling of all key exposures with relevant confounders jointly within a single unified statistical model and explicitly accounted for the clustering and repeated-measures nature of the data thus reducing the likelihood of spurious findings arising from inappropriate independence assumptions thus further reducing the risk of Type I error. Lastly, formal power analyses were not undertaken. Given all adjusted models we reported had > 5000 observations from > 200 individuals, had minimum of 10–20 new NRMCEs per variable/category each analyses, and many significant findings demonstrated small effect sizes, we consider the likelihood of type II error is low.

## Conclusions

Previous neck pain, higher worry scores, larger acute flying workloads, more weekly flights, and larger acute and chronic S&C workloads, were factors observed with an increased risk of new NRMCE. In contrast to chronic S&C workloads, chronic flying workloads demonstrated a non-linear relationship, with higher loads offering a reduction in risk of NRMCE. Acute and chronic sleep quality scores demonstrated similar non-linear relationships with NRMCE risk, which may reflect individuals self-governing effort intensity when sleep quality is notably poor. Neck rotation and trunk flexion isometric strength also demonstrated non-linear relationships, with a ceiling identified where values beyond this increased weekly risk of NRMCE. Demographics (including age, gender, cockpit and flying roles, and aircraft), anthropometry measures, neck and trunk ROM, and S&C participation, however, did not affect risk of new NRMCE.

While some findings appear in contrast with popular beliefs, they are indeed supported by research findings in other military populations, and substantiate the advocacy of S&C programs to keep workloads low and be targeted at known risk factors. Importantly, our findings provide crucial support for the design of targeted prevention programs for FJA, ensuring they are both effective and efficient which will in turn optimise operational capability and workforce health.

## Supplementary Information

Below is the link to the electronic supplementary material.

Supplementary Material 1

Supplementary Material 2

## Data Availability

The data sets used for analysis in this study may be provided upon reasonable request pending approval for release from relevant organisations within Australian Defence Organisation.
