# Supplementary material for: Identifying contributory risk factors for neck pain in fast jet aircrew: a prospective cohort study
Source: Int Arch Occup Environ Health. 2025 Aug 14;98(8):707–20. doi: 10.1007/s00420-025-02162-7 (PMC12494672; doi:10.1007/s00420-025-02162-7)
Supplement: Supplementary file 1 — Supplementary Material 1 [file 420_2025_2162_MOESM1_ESM.docx]

**Online resource 1**

**Identifying contributory risk factors for neck pain in fast jet aircrew - A prospective cohort study**

International Archives of Occupational and Environmental Health

James Wallace^1^, Peter Osmotherly^2^, Tim Gabbett^3^, Wayne Spratford^1^, Phil Newman^1^.

^1^ University of Canberra Research Institute for Sport and Exercise (UCRISE), Bruce ACT, Australia.

^2^ School of Health Sciences, The University of Newcastle, Callaghan NSW, Australia.

^3^ Gabbett Performance Solutions, Brisbane QLD Australia.

Correspondence to: [james.wallace@canberra.edu.au](mailto:james.wallace@canberra.edu.au)

**Baseline data collection**

At the start of each reporting period, FJA were asked to undertake baseline testing, including: questionnaire, range of motion (ROM), isometric strength testing of neck and trunk, predicted one-repetition-maximum (1RM) strength testing of compound movements using free-weights, and cardiorespiratory testing using rowing ergometer.

**Questionnaire:**

The questionnaire captured: age, flying role (student, instructor, or frontline), cockpit role (pilot or back seater – i.e. Weapons Systems Officer or Electronic Warfare Officer), total fast jet flying hours, and previous neck pain (past 3 months, past 12 months, and at all in the past). FJA also rated their personal ‘worry’ on a 0-10 scale (0 = not worried at all, and 10 = extremely worried) regarding the risk of neck/back pain flying fast jets, and, the risk that neck/back pain may one day preclude them from flying fast jets.

**Anthropometry:**

Height, mass, and neck circumference were measured using previously published methods (Norton & Olds, 1996).

**Range of motion (ROM):**

Cervical spine ROM for flexion, extension, rotation was measured using a CROM Device (Performance Attainment Associates, USA) using previously published methods (Fletcher & Bandy, 2008), averaging two trials for each direction. Left and right rotation were highly correlated (r=0.72), thus were summated into the singular *neck transverse plane total ROM*. Trunk rotation ROM was measured using a bubble inclinometer (Baseline, USA) using previously published methods (Iveson et al., 2010), averaging two trials for each direction. Left and right rotation were again highly correlated (r=0.76), thus were summated into the singular *trunk transverse plane total ROM*.

**Isometric maximal strength of neck and trunk:**

Isometric maximal strength of the neck and trunk was measured using David Spine Devices (David Health, Finland). The David G140 was used for neck flexion, extension, and lateral flexion; the G160 for neck rotation; the G130 trunk flexion; the G110 for trunk extension; the G150 for trunk lateral flexion; and, the G120 for trunk rotation. Further information on these devices can be found at: https://davidhealth.com/products/ . Following participant setup in the device as per the David Spine Devices User Guide, participants were familiarised with the testing procedure. Three trials were undertaken, whereby the 1^st^ was 50% effort, followed by two maximal efforts. Sixty seconds rest occurred between trials. For each trial, a 5-second isometric test was undertaken whereby the participant built their force over the first two seconds followed by a maximal effort in the remaining three seconds. A graphical trace/output of each test was visually inspected to ensure optimal effort. The peak force attained from the two maximal trials was used in the analyses of this study. Left and right rotation were both highly correlated in the neck (r=0.91) and trunk (r=0.92), as were left and right lateral flexion in the neck (r=0.93) and trunk (r=0.94), thus the Peak of Left and right were averaged into the singular *isometric neck rotation strength*, *isometric neck lateral flexion strength*, *isometric trunk rotation strength*, and *isometric trunk lateral flexion strength*. Absolute (kg of force produced) and relative (divided by body mass – kg of force produced per kg of body mass) strength values were both analysed independently.

Where possible, testing was not undertaken with individuals when they had flights scheduled later that day.

**Gym-based testing:**

Gym-based testing was conducted by the RAAF Physical Training Instructors and strength & conditioning (S&C) coaches and was conducted over a two day period. Day 1 included: predicted 1RM performance on the back squat and bench press, and cardiorespiratory testing for the 500m row. Day two included: predicted 1RM performance on the deadlift and pull-up, and cardiorespiratory testing for the 5min row.

Predicted 1RM testing was conducted for the squat, dead lift, bench press, and pull-up. Following a general warmup, 5 warmup sets were performed, increasing from 5-reps with barbell only, up to 2 reps at 90% of target weight with 90-120secs rest between sets. A final test set occurred with the aim of reaching 3-5 repetition maximum (3-5RM), and 1RM performance was calculated using the weights achieved on the 3-5RM testing using the conversion equations of Baechle & Earle (Baechle, 2000). Absolute (kg of force produced) and relative (divided by body mass – kg of force produced per kg of body mass) strength values were both analysed independently.

Cardiorespiratory testing was conducted on a Concept2 Model D rowing ergometer (Concept2, USA) for maximal performance on a 500m row and a 5-minute row. Following general warm-up, five 30second sets were performed on the rower progressing from an easy row to 10 strokes holding the goal pace to be achieved in the relevant test. Following a minimum of 2min rest, the test was undertaken where maximal performance on 500m row or 5minute row was undertaken. The time for the 500m row was recorded in seconds, and the distance for the 5minute row was recorded in metres.

Where possible, testing was not undertaken with individuals when they had flights scheduled later that day.

**Weekly data collection**

**Internal workloads:**

Flying internal workloads were captured using session rating of perceived exertion (sRPE) (Foster, 1998; Foster et al., 2001), where FJA were asked rate the global intensity of each sortie using sRPE on a 0-10 rating scale where 0 represented ‘*rest*’ and 10 represented ‘*maximal*’ (Foster, 1998; Foster et al., 2001).

Previous research in sporting settings have sought an sRPE rating by asking ‘*how was your workout*’ (Foster, 1998; Foster et al., 2001; Soligard et al., 2016). FJA however, undertake a range of mission types with a large range of flying demands, for example, air-to-surface, instrument flying (IF) and specific electronic warfare missions may be characterised by high cognitive and technical demands yet low +Gz and thus low physical demands, whereas visual range air-to-air missions (eg. basic fighter manoeuvring – BFM, and air combat manoeuvering - ACM) are characterised by high physical demands due to repeated exposure to high +Gz (and thus repeated anti-G straining manoeuvres) and high neck/trunk demands as they repeatedly scan out the back of, and above, their cockpits to maintain situational awareness. Previous feedback from RAAF FJA was that the above wording of the question would cause cognitive or technical aspects to predominate FJA’s RPE rating, thus we modified the question to ‘*overall, how physically demanding was the flight*’. Further feedback also reinforced that some mission types had high +Gz (thus high physical demands) and high neck demands as FJA had to repeatedly scan out the back and above their cockpits under high +Gz (e.g. defensive BFM), whereas other similarly physically demanding missions did not require such scanning and thus had much lower demands on their neck (e.g. offensive BFM). We therefore elected to capture a second sRPE by asking ‘*overall, how demanding on your neck was the flight*’. The sRPE ratings were then multiplied by the sortie duration (in minutes), as captured by RAAF’s flight resource and scheduling system, to provide two flying workload scores in arbitrary units (AU) – flight physical workload and flight neck workload. All flight workloads between Monday and Sunday of each week were summed to provide a weekly score – ‘*acute flying physical workload*’ and ‘*acute flying neck workload*’. Additionally, *chronic flying physical workload* and *chronic flying neck workload* were quantified by summing the respective workloads from the current week and the previous three weeks. FJA were encouraged to enter their session RPE’s following each flight using a custom flight log using Smartabase smartphone app and online software (Fusion Sport, Australia).

For consistency, S&C internal workloads were similarly calculated for *acute S&C physical workload* and *acute S&C neck workload*, *chronic S&C physical workload* and *chronic S&C neck workload*. FJA were encouraged to enter their session RPE’s and session duration following each S&C session they undertook (including RAAF FJA specific sessions [see below] and their own self-directed sessions) using a custom S&C log using Smartabase smartphone app and online software (Fusion Sport, Australia). Combined internal workload scores were also created by summating the respective flying and S&C internal workloads: *acute combined physical workload* and *acute combined neck workload*, *chronic combined physical workload* and *chronic combined neck workload*.

**Psychosocial stress-recovery (well-being):**

Aspects relating to psychosocial stress-recovery (well-being) were measured using four subscales from the Recovery-Stress Questionnaire – Sport (RESTQ-S) (Kellmann & Kallus, 2001): fatigue, general wellbeing, sleep quality, and fitness/injury. These subscales were chosen based upon their previous demonstrated relationship with injury (Brink et al., 2010; Laux et al., 2015; van der Does et al., 2017), responsiveness to changes in training loads (Saw et al., 2016), the tool (and its subscales) being one of the few to have undergone rigorous psychometric evaluation (Kellmann & Kallus, 2001), and being recommend in the evidence (Jones et al., 2017).

A disturbed breaks subscale had previously been considered for inclusion, but was excluded following feedback from RAAF FJA that the related questions were not relatable or reflective of the FJA working environment. FJA were asked to score 10 questions (as per RESTQ-S 52) regarding ‘*the past three days and nights*’ using a 0-6 scale where 0 represented ‘*never*’ and 6 represented ‘*always*’. Scores for the questions pertaining to each subscale were averaged to provide a single score, with scores for questions regarding a positive statement inverted so that low scores all reflected an ideal response (eg. better sleep and wellbeing, lower fatigue, and low vulnerability to injury). The 10 questions are displayed in the below table, however the order of questions was randomised in the Smartabase log as per the original RESTQ-S questionnaires (Kellmann & Kallus, 2001). FJA were encouraged to complete the questions once per week using a custom wellbeing log using Smartabase smartphone app and online software (Fusion Sport, Australia). An acute and chronic score for each was based upon the scores in a given week, and the average of acute scores over a four-week period respectively.

| **Table A1 -** Psychosocial stress-recovery (well-being) questions | | |
| --- | --- | --- |
| **Subscale** | **Question** | **If scores were inverted** |
| Fatigue | In the past 3 days and nights,  **… I was tired from work** |  |
|  | In the past 3 days and nights,  **… I was dead tired after work** |  |
| General well-being | In the past 3 days and nights,  **… I was in good spirits** | Yes |
|  | In the past 3 days and nights,  **… I was in a good mood** | Yes |
| Sleep quality | In the past 3 days and nights,  **… I had a satisfying sleep** | Yes |
|  | In the past 3 days and nights,  **… I slept restlessly** |  |
| Fitness/injury | In the past 3 days and nights,  **… parts of my body were aching** |  |
|  | In the past 3 days and nights,  **… my muscles felt stiff or tense during performance** |  |
|  | In the past 3 days and nights,  **… I had muscle pain after performance** |  |
|  | In the past 3 days and nights,  **… I felt vulnerable to injuries** |  |
| ***Note:*** *order of questions was randomized as was done in the original RESTQ-S questionnaires* | | |

**Strength and Conditioning (S&C) programs:**

At the time of this study, RAAF had two S&C FJA-specific programs which FJA were encouraged to participate: a gym-based program (gym program) and a targeted neck and back strengthening program using David equipment (David Health, Finland) (David program). FJA were encouraged to undertake both programs 2x per week, however, the gym program was able to accommodate increased frequencies for those who wished to do so. Experience demonstrated FJA commonly undertook one such program based on their own preference, thus a hybrid program (essentially the gym program plus the neck exercises from the David program) was encouraged at some FJA sites to ensure FJA received the benefits of both programs.

The gym program consisted of free weight strength exercises and cardiorespiratory (aerobic/anaerobic) conditioning. The strength training component was characterised by:

- Six main lifts/exercises: lower body push (squat), lower body pull (deadlift), upper body vertical pull (pull-up), upper body vertical push (push press, overhead press), trunk rotation, and trunk anti-rotation (isometric rotation);
- Prescription: block periodised; percentage-based training based upon predicted 1RM testing (from 3-5RM testing) (except for rotation and anti-rotation exercises); volume range 3-5 sets of 4-12 reps; intensity range 60-90+% 1RM.

The cardiorespiratory training component was characterised by:

- Modalities: rowing ergometer, assault bike;
- Prescription: block periodised; percentage-based training based upon 5-minute maximal rowing effort; volume 4-18 minutes of 15-to 300-second sets; intensity 80-120+% of 5-min max rowing effort.

The David program consisted of strength training using David Spine Devices (David Health, Finland) which were pin loaded machines allowing targeted strength training through each plane (sagittal, frontal, and transverse) of the neck and trunk.

- Modalities: The David G140 was used for neck flexion, extension, and lateral flexion; the G160 for neck rotation; the G130 trunk flexion; the G110 for trunk extension; the G150 for trunk lateral flexion; and, the G120 for trunk rotation.
- Prescription: block periodised; autoregulated training based upon individuals perceived repetitions in reserve and RPE; volume range 1-3 sets of 6-20 reps; intensity 5-8/10 RPE (based 0-10 RPE scale).

Both programs attempted to match training to the individual (including their training history), and current (and upcoming) flying phase/s (i.e. lower intensity during more demanding flying phases such as BFM and ACM).

* Note, these programs were in place during the study period (2019-2020) and may not reflect current RAAF FJA S&C programs.

**Participation** in the gym program was captured by relevant S&C staff present in the facilities and using custom S&C log using Smartabase (FusionSport, Australia), and David program participation was captured by the software within the David equipment. Participation in a given week was considered to have occurred when an individual completed two sessions (on separate days) of a given program in a week (e.g. 1x session of the David program and 1x session of the gym program in a given week was not considered to have participated twice or more in a given program). Participation was evaluated for each program over 8 and 12 week periods.

**R-packages used**

| **Table A2** – R-packages used in this study |
| --- |
| **Package** |
| Tidyverse |
| Mice |
| Sjmisc |
| Ggthemes |
| ggplot2 |
| Openxlsx |
| Rms |
| lme4 |
| merTools |
| sjPlot |
| Ggeffects |
| clubSandwich |
| Car |
| Psych |
| Optimx |
| Webshot |

**Addressing convergence issues**

**Optimisers used to address convergence issues in multilevel logistic regression:**

Convergence issues were addressed by scaling continuous predictors (by 10, 100, or 1000 where appropriate), substituting the optimisers as per the below table, and reconsidering the inclusion of any random slopes (Winter, 2019).

| **Table A3** – Optimisers used |
| --- |
| **Relevant R code for optimisers used** |
| … control = glmerControl(optimizer = "**bobyqa**")) |
| … control = glmerControl(optimizer ="**Nelder_Mead**") |
| … control = glmerControl(optimizer ='optimx', optCtrl=list(method=**'L-BFGS-B**'))) |
| … control = glmerControl(optimizer ='optimx', optCtrl=list(method='**nlminb**'))) |

**Reference list for Online Resource 1**

Baechle, T., Earle, R, and Wathen, D. (2000). Resistance training. In T. Baechle, and Earle, R (Ed.), *Essentials of Strength Training and Conditioning* (2nd ed., pp. 395-425). Human Kinetics.

Brink, M. S., Visscher, C., Arends, S., Zwerver, J., Post, W. J., & Lemmink, K. A. (2010). Monitoring stress and recovery: new insights for the prevention of injuries and illnesses in elite youth soccer players. *British Journal of Sports Medicine*, *44*(11), 809-815.

Fletcher, J. P., & Bandy, W. D. (2008). Intrarater reliability of CROM measurement of cervical spine active range of motion in persons with and without neck pain. *Journal of Orthopaedic and Sports Physical Therapy*, *38*(10), 640-645.

Foster, C. (1998). Monitoring training in athletes with reference to overtraining syndrome. *Medicine and Science in Sports and Exercise*, *30*(7), 1164-1168.

Foster, C., Florhaug, J. A., Franklin, J., Gottschall, L., Hrovatin, L. A., Parker, S., Doleshal, P., & Dodge, C. (2001). A new approach to monitoring exercise training. *The Journal of Strength & Conditioning Research*, *15*(1), 109-115.

Iveson, B. D., McLaughlin, S. L., Todd, R. H., & Gerber, J. P. (2010). Reliability and exploration of the side-lying thoraco-lumbar rotation measurement (STRM). *North American journal of sports physical therapy: NAJSPT*, *5*(4), 201.

Jones, C. M., Griffiths, P. C., & Mellalieu, S. D. (2017). Training load and fatigue marker associations with injury and illness: a systematic review of longitudinal studies. *Sports Medicine*, *47*, 943-974.

Kellmann, M., & Kallus, K. W. (2001). *Recovery-stress questionnaire for athletes: User manual*. Human Kinetics.

Laux, P., Krumm, B., Diers, M., & Flor, H. (2015). Recovery–stress balance and injury risk in professional football players: a prospective study. *Journal of Sports Sciences*, *33*(20), 2140-2148.

Norton, K., & Olds, T. (1996). *Anthropometrica: a textbook of body measurement for sports and health courses*. UNSW press.

Saw, A. E., Main, L. C., & Gastin, P. B. (2016). Monitoring the athlete training response: subjective self-reported measures trump commonly used objective measures: a systematic review. *British Journal of Sports Medicine*, *50*(5), 281-291.

Soligard, T., Schwellnus, M., Alonso, J.-M., Bahr, R., Clarsen, B., Dijkstra, H. P., Gabbett, T., Gleeson, M., Hägglund, M., & Hutchinson, M. R. (2016). How much is too much?(Part 1) International Olympic Committee consensus statement on load in sport and risk of injury. *British Journal of Sports Medicine*, *50*(17), 1030-1041.

van der Does, H. T. D., Brink, M. S., Otter, R. T. A., Visscher, C., & Lemmink, K. A. P. M. (2017). Injury risk is increased by changes in perceived recovery of team sport players. *Clinical Journal of Sport Medicine*, *27*(1), 46-51.

Winter, B. (2019). *Statistics for linguists: An introduction using R*. Routledge.
