# Supplementary material for: Identifying contributory risk factors for neck pain in fast jet aircrew: a prospective cohort study
Source: Int Arch Occup Environ Health. 2025 Aug 14;98(8):707–20. doi: 10.1007/s00420-025-02162-7 (PMC12494672; doi:10.1007/s00420-025-02162-7)
Supplement: Supplementary file 2 — Supplementary Material 2 [file 420_2025_2162_MOESM2_ESM.docx]

**Online resource 2**

**Identifying contributory risk factors for neck pain in fast jet aircrew - A prospective cohort study**

International Archives of Occupational and Environmental Health

James Wallace^1^, Peter Osmotherly^2^, Tim Gabbett^3^, Wayne Spratford^1^, Phil Newman^1^.

^1^ University of Canberra Research Institute for Sport and Exercise (UCRISE), Bruce ACT, Australia.

^2^ School of Health Sciences, The University of Newcastle, Callaghan NSW, Australia.

^3^ Gabbett Performance Solutions, Brisbane QLD Australia.

Correspondence to: [james.wallace@canberra.edu.au](mailto:james.wallace@canberra.edu.au)

| **Table 4** - Findings for baseline data collection - Simple models | | | | | | |
| --- | --- | --- | --- | --- | --- | --- |
| **Factor** | **Model specifications** | **Observations (n FJA)** | **New NRMC episodes** | **Observation mean (SD), or # per category** | **OR (95% CI) ^a^** | ***p*** |
| **Demographics** |  |  |  |  |  |  |
| Age (per year) | RCS-3 (2RI) | 10127 (252) | 277 | 33.1 (8.2) | 1.09 (1.01-1.18) | **0.034 *** |
|  |  |  |  |  | 0.88 (0.77-1.00) | **0.05 *** |
| Gender: - Male | IO (2RI) | 10837 (279) | 320 | 10285 | 1.00 ^b^ |  |
| - Female |  |  |  | 552 | 1.75 (0.75-4.10) | 0.195 |
| Flying role: - Student | IO (2RI) | 10837 (279) | 320 | 1580 | 1.00 ^b^ |  |
| - Front Line |  |  |  | 6343 | 1.31 (0.73-2.36) | 0.362 |
| - Instructor |  |  |  | 2914 | 1.59 (0.83-3.04) | 0.158 |
| Cockpit role: - Pilot | IO (2RI) | 10786 (276) | 317 | 8938 | 1.00 ^b^ |  |
| - Back seater |  |  |  | 1848 | 0.94 (0.55-1.61) | 0.819 |
| Current aircraft: - F/18F | IO (2RI) | 10837 (279) | 320 | 2406 | 1.00 ^b^ |  |
| - EA-18G |  |  |  | 1290 | 0.86 (0.38-1.97) | 0.722 |
| - F-35 |  |  |  | 735 | 0.86 (0.37-1.97) | 0.721 |
| - F/A-18C |  |  |  | 2878 | 1 (0.53-1.88) | 0.992 |
| - Hawk |  |  |  | 3528 | 0.82 (0.46-1.49) | 0.522 |
| **Questionnaire** |  |  |  |  |  |  |
| Neck pain - past 3mo: - No | IO (1RI) | 6336 (223) | 156 | 5619 | 1.00 ^b^ |  |
| - Yes |  |  |  | 717 | 2.65 (1.61-4.36) | **<0.001*** |
| Neck pain - past 12mo: - No | IO (2RI) | 6727 (225) | 164 | 5047 | 1.00 ^b^ |  |
| - Yes |  |  |  | 1680 | 2.65 (1.74-4.04) | **<0.001*** |
| Neck pain - past at all: - No | I&S (2RI) | 7965 (230) | 217 | 3824 | 1.00 ^b^ |  |
| - Yes |  |  |  | 4141 | 2.67 (1.35-5.29) | **0.005 *** |
| Total fighter hours (per 100hrs) | RCS-4 (2RI) | 9831 (239) | 265 | 12.92 (13.91) | 1.21 (1.05-1.40) | **0.01 *** |
|  |  |  |  |  | 0.13 (0.02-0.77) | **0.024 *** |
|  |  |  |  |  | 18.8 (1.4-256.6) | **0.028 *** |
| Worry - Risk of neck/back pain flying fighters (per 1 unit) | IO (1RI) | 6242 (223) | 155 | 3.16 (2.19) | 1.2 (1.10-1.31) | **<0.001*** |
| Worry - Risk neck/back pain may preclude you from fighters in future (per 1 unit) | IO (1RI) | 6242 (223) | 155 | 3.14 (2.47) | 1.17 (1.08-1.27) | **<0.001*** |
| **Anthropometry** |  |  |  |  |  |  |
| Height (per 10cm) | IO (2RI) | 7654 (250) | 211 | 18.01 (0.59) | 0.71 (0.49-1.03) | 0.075 |
| Body mass (per 10kg) | IO (1RI) | 7735 (254) | 214 | 8.12 (1.04) | 0.79 (0.62-1.00) | **0.048 *** |
| Neck circumference (per cm) | I&S (2RI) | 7009 (238) | 194 | 38.17 (2.55) | 0.86 (0.78-0.94) | **0.001 *** |
| **Range of motion** |  |  |  |  |  |  |
| Neck flexion (per 10°) | IO (2RI) | 7044 (237) | 193 | 5.86 (0.89) | 0.95 (0.76-1.20) | 0.673 |
| Neck extension (per 10°) | IO (2RI) | 7044 (237) | 193 | 7.52 (1.01) | 0.89 (0.73-1.09) | 0.257 |
| Neck transverse plane total (per 10°) | IO (2RI) | 7044 (237) | 193 | 13.96 (1.57) | 0.88 (0.77-1.00) | 0.053 |
| Trunk transverse plane total (per 10°) | IO (2RI) | 6968 (236) | 193 | 11.39 (2.17) | 1.01 (0.92-1.11) | 0.819 |
| **Isometric spinal strength – Absolute** |  |  |  |  |  |  |
| Neck flexion (per 10kg) | RCS-4 (1RI) | 7207 (249) | 205 | 3.13 (1.04) | 0.43 (0.22-0.84) | **0.014 *** |
|  |  |  |  |  | 8.2 (0.7-100.4) | 0.099 |
|  |  |  |  |  | 0 (0.00-12.60) | 0.163 |
| Neck extension (per 10kg) | IO (1RI) | 7242 (250) | 205 | 5.83 (1.45) | 0.96 (0.84-1.10) | 0.601 |
| Neck rotation (per 10kg) | RCS-3 (1RI) | 7134 (249) | 205 | 1.6 (0.49) | 0.37 (0.16-0.86) | **0.02 *** |
|  |  |  |  |  | 2.67 (1.03-6.93) | **0.043 *** |
| Neck lateral flexion (per 10kg) | IO (1RI) | 7232 (250) | 205 | 4.58 (1.23) | 0.88 (0.76-1.03) | 0.122 |
| Trunk flexion (per 10kg) | RCS-4 (1RI) | 7232 (250) | 204 | 19.92 (5.55) | 0.81 (0.70-0.94) | **0.006 *** |
|  |  |  |  |  | 1.86 (1.08-3.21) | **0.025 *** |
|  |  |  |  |  | 0.16 (0.03-0.96) | **0.044 *** |
| Trunk extension (per 10kg) | IO (1RI) | 7205 (249) | 198 | 29.92 (8.15) | 0.98 (0.95-1.00) | 0.063 |
| Trunk rotation (per 10kg) | IO (1RI) | 7223 (250) | 204 | 19.63 (5.49) | 0.98 (0.94-1.01) | 0.156 |
| Trunk lateral flexion (per 10kg) | IO (1RI) | 7252 (250) | 204 | 20.78 (5.99) | 0.99 (0.96-1.02) | 0.378 |
| **Isometric spinal strength – Relative** |  |  |  |  |  |  |
| Neck flexion (kg per kg BM) | IO (1RI) | 7196 (248) | 205 | 0.39 (0.12) | 0.23 (0.05-1.13) | 0.071 |
| Neck extension (kg per kg BM) | IO (1RI) | 7231 (249) | 205 | 0.72 (0.16) | 1.02 (0.31-3.35) | 0.979 |
| Neck rotation (kg per kg BM) | IO (1RI) | 7123 (248) | 205 | 0.2 (0.06) | 0.17 (0.01-4.85) | 0.298 |
| Neck lateral flexion (kg per kg BM) | IO (1RI) | 7221 (249) | 205 | 0.57 (0.14) | 0.44 (0.12-1.68) | 0.231 |
| Trunk flexion (kg per kg BM) | I&S (1RI) | 7221 (249) | 204 | 2.44 (0.56) | 0.68 (0.39-1.19) | 0.176 |
| Trunk extension (kg per kg BM) | IO (1RI) | 7194 (248) | 198 | 3.68 (0.88) | 0.85 (0.68-1.06) | 0.152 |
|  |  |  |  |  |  |  |
| Trunk rotation (kg per kg BM) | IO (1RI) | 7212 (249) | 204 | 2.41 (0.58) | 0.85 (0.62-1.17) | 0.315 |
| Trunk lateral flexion (kg per kg BM) | IO (1RI) | 7241 (249) | 204 | 2.56 (0.65) | 0.93 (0.69-1.24) | 0.607 |
| **Gym-based testing – Absolute** |  |  |  |  |  |  |
| Pull-up (per 10kg) | IO (1RI) | 2862 (110) | 69 | 10.33 (1.69) | 0.99 (0.80-1.23) | 0.948 |
| Bench press (per 10kg) | IO (1RI) | 3249 (127) | 73 | 8.47 (1.96) | 1.08 (0.92-1.27) | 0.366 |
| Deadlift (per 10kg) | IO (1RI) | 3032 (119) | 73 | 13.08 (3.2) | 1 (0.90-1.11) | 0.961 |
| Squat (per 10kg) | IO (1RI) | 3182 (124) | 72 | 10.58 (2.38) | 1.03 (0.90-1.17) | 0.679 |
| 5min row (per 100m) | IO (1RI) | 2868 (114) | 62 | 13.35 (0.83) | 0.9 (0.60-1.36) | 0.628 |
| 500m row (per 10secs) | IO (1RI) | 2999 (117) | 68 | 9.58 (0.74) | 1.05 (0.66-1.67) | 0.832 |
| **Gym-based testing – Relative** |  |  |  |  |  |  |
| Pull-up (kg per kg BM) | IO (1RI) | 2862 (110) | 69 | 1.27 (0.14) | 1.38 (0.12-16.05) | 0.799 |
| Bench press (kg per kg BM) | IO (1RI) | 3249 (127) | 73 | 1.03 (0.2) | 3.03 (0.62-14.83) | 0.172 |
| Deadlift (kg per kg BM) | IO (1RI) | 3032 (119) | 73 | 1.6 (0.34) | 1.02 (0.37-2.80) | 0.974 |
| Squat (kg per kg BM) | IO (1RI) | 3182 (124) | 72 | 1.3 (0.25) | 1.56 (0.46-5.26) | 0.475 |
| * p <0.05  ^a^ For categorical data Odds Ratio provided for given category compared to reference category; For continuous data Odds Ratio provided per unit increase as noted in the factor column.  ^b^ Reference category.  1RI, one random intercept only (individual FJA); 2RI, two random intercepts (cross-nested: individual FJA and squadron for given semester); 3mo, 3 months; 12mo, 12 months; BW, body mass; IO, random intercept only; I&S, random intercept and random slope; OR, odds ratio; RCS-3, restricted cubic splines using three knots; RCS-4, restricted cubic splines using four knots; SD, standard deviation; NRMC, neck related musculoskeletal complaint; WL, workload | | | | | | |

| **Table 5** - Findings for weekly data collection – Simple models | | | | | | |
| --- | --- | --- | --- | --- | --- | --- |
|  | **Model specifications** | **Observations (n FJA)** | **New NRMC episodes** | **Observation mean (SD)** | **OR (95% CI) ^b^** | ***p*** |
| **External Flying Workload** |  |  |  |  |  |  |
| Weekly number of flights (per 1 flight) | IO (2RI) | 10837 (279) | 320 | *Not provided* ^a^ | 1.12 (1.04-1.20) | **0.002 *** |
| Weekly total flight time (per 1 hour) | IO (2RI) | 10837 (279) | 320 | *Not provided* ^a^ | 1.03 (0.98-1.08) | 0.242 |
| **Internal Flying Workload** |  |  |  |  |  |  |
| Acute flying neck workload (per 1000 units) | IO (2RI) | 10837 (279) | 320 | 0.39 (0.45) | 1.53 (1.22-1.92) | **<0.001*** |
| Acute flying physical workload (per 1000 units) | IO (2RI) | 10837 (279) | 320 | 0.46 (0.51) | 1.42 (1.15-1.76) | **0.001 *** |
| Chronic flying neck workload (per 1000 units) | IO (2RI) | 10837 (279) | 320 | 1.51 (1.21) | 1.13 (1.03-1.25) | **0.01 *** |
|  |  |  |  |  |  |  |
| Chronic flying physical workload (per 1000 units) | IO (2RI) | 10837 (279) | 320 | 1.8 (1.45) | 1.1 (1.01-1.20) | **0.028 *** |
|  |  |  |  |  |  |  |
| **Internal S&C Workload** |  |  |  |  |  |  |
| Acute S&C neck workload (per 1000 units) | I&S (2RI) | 10837 (279) | 320 | 0.27 (0.32) | 2.16 (1.25-3.71) | **0.005 *** |
| Acute S&C physical workload (per 1000 units) | I&S (2RI) | 10837 (279) | 320 | 0.58 (0.63) | 1.28 (0.90-1.82) | 0.171 |
| Chronic S&C neck workload (per 1000 units) | I&S (2RI) | 10837 (279) | 320 | 1.05 (1.08) | 1.3 (1.09-1.55) | **0.003 *** |
| Chronic S&C physical workload (per 1000 units) | RCS-3 (2RI) | 10837 (279) | 320 | 2.25 (2.14) | 0.86 (0.70-1.05) | 0.129 |
|  |  |  |  |  | 1.31 (1.02-1.68) | **0.032 *** |
| **Internal combined workload** |  |  |  |  |  |  |
| Acute combined neck workload (per 1000 units) | IO (2RI) | 10837 (279) | 320 | 0.65 (0.58) | 1.44 (1.19-1.74) | **<0.001 *** |
| Acute combined physical workload (per 1000 units) | I&S (2RI) | 10837 (279) | 320 | 1.03 (0.85) | 1.52 (1.24-1.86) | **<0.001 *** |
| Chronic combined neck workload (per 1000 units) | IO (2RI) | 10837 (279) | 320 | 2.56 (1.79) | 1.11 (1.03-1.19) | **0.004 *** |
|  |  |  |  |  |  |  |
| Chronic combined physical workload (per 1000 units) | IO (2RI) | 10837 (279) | 320 | 4.05 (2.76) | 1.06 (1.01-1.12) | **0.011 *** |
| **S&C Participation** |  |  |  |  |  |  |
| David 8-week participation (per week) ^c, d^ | IO (2RI) | 8583 (279) | 243 | 1.59 (2.17) | 0.99 (0.92-1.07) | 0.878 |
| David 12-week participation (per week) ^c, d^ | IO (1RI) | 6333 (271) | 170 | 2.3 (2.95) | 0.93 (0.87-1.00) | **0.049 *** |
| FF Gym 8-week participation (per week) ^c, d^ | IO (2RI) | 8583 (279) | 243 | 2.79 (2.68) | 0.96 (0.90-1.02) | 0.174 |
| FF Gym 12-week participation (per week) ^c, d^ | IO (1RI) | 6333 (271) | 170 | 4.07 (3.75) | 0.95 (0.91-1.01) | 0.08 |
| **Wellbeing** |  |  |  |  |  |  |
| Acute fatigue (per 1 unit) | RCS-3 (1RI) | 9508 (276) | 265 | 1.14 (1.08) | 1.78 (1.16-2.71) | **0.008 *** |
|  |  |  |  |  | 0.57 (0.36-0.91) | **0.02 *** |
| Chronic fatigue (per 1 unit) | IO (1RI) | 8655 (269) | 234 | 1.13 (0.92) | 1.29 (1.09-1.54) | **0.004 *** |
| Acute general wellbeing (per 1 unit) | RCS-3 (1RI) | 9494 (276) | 264 | 1.76 (1.22) | 1.42 (0.93-2.17) | 0.108 |
|  |  |  |  |  | 0.67 (0.45-0.98) | **0.039 *** |
| Chronic general wellbeing (per 1unit) | IO (1RI) | 8636 (269) | 233 | 1.76 (1.13) | 1.07 (0.92-1.26) | 0.374 |
| Acute sleep quality (per 1 unit) | I&S (1RI) | 9496 (276) | 265 | 1.97 (1.20) | 1.07 (0.94-1.21) | 0.297 |
|  |  |  |  |  |  |  |
| Chronic sleep quality (per 1 unit) | IO (1RI) | 8638 (269) | 234 | 1.98 (1.06) | 1.24 (1.05-1.46) | **0.013 *** |
|  |  |  |  |  |  |  |
| Acute fitness/injury (per 1 unit) | I&S (1RI) | 9630 (276) | 270 | 2.08 (0.62) | 1.25 (0.91-1.70) | 0.164 |
| Chronic fitness/injury (per 1 unit) | IO (1RI) | 8785 (270) | 240 | 2.08 (0.51) | 1.57 (1.17-2.09) | **0.002 *** |
| * p <0.05  ^a^ Not provided for security reasons.  ^b^ Odds Ratio provided per unit increase as noted in the factor column.  ^c^ A week of participation indicates one week where two sessions of a given program were undertaken (e.g. a participation value of 8 indicates an individual has performed a given program twice per week on 8 separate weeks).  ^d^ Individuals had to be present in the previous 7 weeks, or previous 11 weeks, AND the current week for their data to be included in these analyses.  1RI, one random intercept only (individual FJA); 2RI, two random intercepts (cross-nested: individual FJA and squadron for given semester); 12mo, 12 months; IO, random intercept only; I&S, random intercept and random slope; OR, odds ratio; RCS-3, restricted cubic splines using three knots; RCS-4, restricted cubic splines using four knots; SD, standard deviation; NRMC, neck related musculoskeletal complaint. | | | | | | |
